# Strengthening Mechanism for the Mechanical Properties of Cement-Based Materials after Internal Nano-SiO_2_ Production

**DOI:** 10.3390/nano12224047

**Published:** 2022-11-17

**Authors:** Jie Zhang, Yongsheng Ji, Zhanguo Ma, Jianwei Cheng, Shengnan Xu, Zhishan Xu, Zhongzhe Zhang

**Affiliations:** 1 State Key Laboratory for Geomechanics and Deep Underground Engineering, China University of Mining and Technology, Xuzhou 221116, China; 2 Xuzhou College of Industrial Technology, Xuzhou 221140, China; 3 Jiangsu Key Laboratory Environmental Impact and Structural Safety in Engineering, China University of Mining and Technology, Xuzhou 221116, China

**Keywords:** nano-SiO_2_ precursor solution (NSPS), internal production, cement-based materials, compressive strength, strengthening mechanism

## Abstract

This study focuses on overcoming the agglomeration issue of nano-SiO_2_ powder in cement, facilitating the strengthening mechanism of cement-based materials. A nano-SiO_2_ precursor solution (NSPS) was added to cement-based materials to replace nano-SiO_2_ powder. The influencing laws of the alkalinity and dosage of the NSPS on the mechanical properties of cement were investigated. Further, the strengthening mechanism of the mechanical properties of cement-based materials after internal nano-SiO_2_ production was analysed. The results show that (1) when the alkalinity of the precursor solution is a weak acid (pH = 6), the compressive strength of cement-based materials after internal nano-SiO_2_ production is 25%~36% higher than that of pure cement-based materials and 16%~22% higher than that of cement-based materials with silica fume; (2) when the solid content of SiO_2_ in the current displacement solution is about 0.16% of the cement mass, the compressive strength of the prepared cement-based material is the highest. With the continuous increase in the solid content of SiO_2_ in the precursor solution, the compressive strength of cement-based materials after internal nano-SiO_2_ production decreases but is always greater than the compressive strength of the cement-based material mixed with nano-SiO_2_ micro powder. According to a microstructural analysis, nano-SiO_2_ particles that precipitate from the precursor solution can facilitate the hydration process of cement and enrich the gel products formed on the cement particle surface. In addition, new network structures among cement particles are formed, and precipitated nano-SiO_2_ particles fill in the spaces among these cement particles as crystal nuclei to connect the cement particles more tightly and compact the cement-based materials. This reinforces the mechanical properties of cement-based materials.

## 1. Introduction

Nano-SiO_2_ (NS) is a typical nanomaterial used for the modification of cement-based materials; its diameter is about 10–100 nm. With a smaller particle size and higher pozzolanic activity than silica powder, nano-SiO_2_ can be used in fillers, volcanic ash, and seeds more effectively [1,2,3,4,5]. It can also react quickly with calcium hydroxide to generate calcium silicate hydrate, facilitating hydration reactions of gel materials and compacting their microstructure [6,7]. This improves the structure and performance of cement mortar and cement mortar–aggregate interfaces, facilitating improvements to the mechanical properties, durability, and impermeability of concrete [8,9,10,11,12,13]. These advantages have led to nano-SiO_2_ being widely used in the modification of cement-based composites [14,15]. The results of [16,17] show that nano-SiO_2_ can improve the early strength of concrete but has little impact on the later strength. The study found that after the incorporation of about 3% nano-SiO_2_ to replace silica ash, the strength of cement stone, cement sand, and concrete was improved compared with the use of silica ash (10%) alone. The strength of cement stone increased by about 20%, the strength of cement sand increased by about 10%, and the increase in concrete strength was less than 10%. Su Yong [18] believes that the excessive incorporation of nano-SiO_2_ will cause a sharp increase in water demand and nano-SiO_2_ dispersion deterioration, causing the strength of concrete to decrease. Based on the above analysis, it can be seen that although nanomaterials have a certain effect on enhancing cement-based materials, the improvement effect is not substantial, which is far from people’s expectations for nanomaterials.

Based on previous research by the authors [19], an NSPS can be prepared using the liquid-phase method. NSPS solutions are acid solutions in which nano-SiO_2_ particles have not yet precipitated. After it is added to cement mortar, SiO_2_ precipitates as nanoparticles that may disperse uniformly in cement mortar. This preparation method not only requires simple technology and is low-cost but can also easily mitigate the agglomeration issue affecting nano-SiO_2_ [20]. The aforementioned studies only focus on the preparation technique of the precursor solution, in addition to the synthesis and precipitation of nano-SiO_2_ in cement-based materials. However, a systematic and in-depth experimental study and mechanism analyses on how cement-based materials participate in the reaction and whether they contribute to the improvement of the mechanical properties of cement-based materials are imperative.

This paper focuses on how an NSPS can enhance the mechanical properties of cement-based materials. With pure cement-based materials and cement-based materials mixed with nano-SiO_2_ mineral powder as reference materials, through the combination of a compressive strength test and scanning electron microscopic (SEM) detection test, this paper studies how an NSPS with different pH values participates in the hydration reaction of cement-based materials; the influence of the solid content of nano-SiO_2_ in the precursor solution on the hydration products; and how the generated hydration products can improve the compressive strength of cement-based materials [2]. Finally, the basic principle of the hydration of cement-based materials generated with nano-SiO_2_ is analysed in the form of a mechanism diagram. The gel products generated by hydration act on the pores between cement particles to enhance the mechanical properties of cement-based materials [20].

## 2. Raw Materials and Test Methods

### 2.1. Raw Materials

(1) Cement

P.O 42.5 cement was produced by ZHONG LIAN Cement Group, Zhonglian, Qingdao, China. Its density was 3.14 g/cm^3^, and the water requirement for normal consistency was 28.0%. The 0.08 mm square hole sieve residue was 1.02%, and the specific surface area was 3300 cm^2^/g. Its specific chemical composition is given in Table 1.

(2) Sodium silicate

Sodium silicate was the silicon source for preparing the nano-SiO_2_ precursor solution based on the liquid-phase method; it is an adhesive in aqueous silicate solutions. The sodium silicate solution was obtained from Zhonglian Cement Group, Zhonglian, Qingdao, China. Its specific physical properties and chemical composition are given in Table 2 and Table 3, respectively.

(3) Acetic acid

The mass fraction of the acetic acid medium used was 99% (analytically pure), and the physical properties of the acetic acid media are given in Table 4.

(4) Nano-SiO_2_ mineral powder

Nano-SiO_2_ powder, an amorphous white powder with a particle size ranging between 1 and 100 nm, was chosen as the control group. It is non-toxic, tasteless, and non-polluting, with a spherical microstructure that looks like a flocculent and networked quasi-granular structure. Its molecular and structural formula is SiO_2_, and it is insoluble in water. The nano-SiO_2_ powder was produced by a company in Shanghai; its specific physical properties are given in Table 5.

### 2.2. Mix Design and Preparation of Specimens

#### 2.2.1. Preparation of NSPS

Sodium silicate solution was dropped into the diluted acetic acid excitant solution using a dropper, during which the mixture was stirred quickly to avoid transient coagulation into flocculates because of excessive local alkalinity. The pH changes were monitored throughout the titration process using an acidimeter until the pH of the solution increased to the target value. The NSPS was prepared following this procedure.

#### 2.2.2. Calculation of Available SiO_2_ Solid Content in the Precursor Solution

The available SiO_2_ solid content in the precursor solution was calculated as follows:

(1) $$m_{{\mathrm{SiO}}_{2}}=m_{2}\times c_{2}\times \left(1-63\%\right)\times 71.6\%$$

The water content in the precursor solution was calculated as:

(2) $$m_{0}=m_{1}\times c_{1}+m_{2}\times c_{2};\;w_{0}=m-m_{0}$$

where *m*_SiO2_ is the available nano-SiO_2_ solid content in the precursor solution (g); *m*_0_ is the mass of solute in the precursor solution (g); *m*_1_ is the mass of acetic acid (g); *c*_1_ is the mass percentage concentration of acetic acid (%); *m*_2_ is the mass of sodium silicate (g); *c*_2_ is the concentration of sodium silicate (%); *m* is the total mass of the precursor solution; and 63% and 71.6% refer to values in Table 2 and Table 3.

#### 2.2.3. Preparation of Cement-Based Specimens through Internal Nano-SiO_2_ Production

According to the GB/T 17671-2021 test method of cement mortar strength (ISO method) [21], the precursor solution prepared in Section 2.2.1 was added during the preparation of cement-based materials. In this process, the mixture was stirred quickly and uniformly to complete the preparation of cement-based materials with internal nano-SiO_2_ production. Then, 40 mm × 40 mm × 160 mm moulds were prepared. All specimens were vibrated on the vibration table for 2 min. Subsequently, the mould mortar specimens were cured for 24 h at 20 ± 2 °C and a relative humidity of ≥90%. Specimens were demoulded after 24 h of casting and then cured at a constant temperature of 21 ± 1 °C and relative humidity of ≥95% for 3 days, 7 days, and 28 days, successively. At least six specimens were prepared for each formula. To prevent the evaporation of water, a plastic film was used to cover the specimens during the solidification and hardening process [22].

#### 2.2.4. Microstructural Analysis

An FEI QUANTA 250 environmental scanning electron microscope (SEM) in USA coupled with an OXFORD Ultim Extreme - Energy Dispersive Spectroscopy (EDS) in Britain were used to examine the morphology of the reaction products formed in the cement-based materials after internal nano-SiO_2_ production. Samples for analysis were 5 mm thick, cut with a diamond precision saw from a freshly fractured piece of the cement-based materials. These samples were immersed in absolute ethyl alcohol to terminate hydration and vacuum-dried at 45 °C. The dried sample was epoxy-impregnated, polished, gold-coated, and examined using SEM in BSE mode. The element compositions were quantitatively analysed through EDX. During fine polishing, an oil-based lubricant was applied to disperse the polishing. These trials were conducted using 28-day mortar samples prepared and cured as described above [23]. 

### 2.3. Research Content

#### 2.3.1. Effects of the Alkalinity of the Precursor Solution on the Compressive Strength of Cement-Based Materials

Cement mortar specimens with the precursor solution were prepared according to the ISO standard of the Cement Mortar Strength Test Method. The compressive strengths of these cement mortar specimens at different ages after internal production were tested by changing the alkalinity of the precursor solution. The effects of the alkalinity of the precursor solution on the mechanical properties of cement-based materials were also studied [24].

Three alkalinity levels (4, 6, and 8) were set for the precursor solution. The precursor solution was prepared at each alkalinity level according to the procedure outlined in Section 2.2.1. The available SiO_2_ solid contents were calculated using Equation (1), obtaining 0.11 g, 0.74 g, and 0.82 g, respectively. The water contents were calculated according to Equation (2) and were found to be 40.88 g, 47.93 g, and 48.85 g, respectively. The specific mixing ratios are given in Table 6. The water mass in Table 6 was calculated according to the total water content (225 g). Precursor solutions with different alkalinity levels were added to the cement mortar to prepare the cement mortar specimens (J1–J3). Further, pure cement-based materials without precursor solutions were prepared as the control group (J0).

For the convenience of comparative analysis, cement mortar specimens with nano-SiO_2_ powder were prepared. The dosage of nano-SiO_2_ powder was consistent with the nano-SiO_2_ solid contents in the precursor solution, which were 0.11 g, 0.74 g, and 0.82 g, respectively.

#### 2.3.2. Effects of Available SiO_2_ Solid Content in the Precursor Solution on the Compressive Strength of Cement-Based Materials

Cement mortar specimens with different SiO_2_ solid contents in their precursor solutions were prepared according to the ISO standard of the Cement Mortar Strength Test Method. The compressive strengths of cement mortar specimens with precursor solutions at different ages after internal production were tested by changing the SiO_2_ solid contents. The effects of the SiO_2_ solid content in the precursor solution on the mechanical properties of cement-based materials were also studied.

The alkalinity of the precursor solution was set to 6, and the mass of the precursor solution was set to 40 g, 60 g, 80 g, and 100 g in different tests. The available SiO_2_ solid content was calculated according to Equation (1), which was divided into four levels: 0.74 g, 1.10 g, 1.47 g, and 1.84 g. The precursor solution at each alkalinity level was prepared according to the process given in Section 2.2.1. The water contents were calculated according to Equation (2), which were found to be 47.93 g, 71.90 g, 95.86 g, and 119.83 g, respectively. The specific mixing ratios are given in Table 7. The precursor solutions with different available SiO_2_ solid contents were added to the cement mortar to prepare their corresponding cement mortar specimens (S1–S4). Furthermore, a pure cement-based material—without a precursor solution—was prepared as the control group (S0).

To facilitate comparative analysis, the dosage of nano-SiO_2_ powder was consistent with the nano-SiO_2_ solid content in the precursor solution at 0.74 g, 1.10 g, 1.47 g, and 1.84 g, respectively.

#### 2.3.3. Microstructural Analysis of Cement-Based Materials after Internal Nano-SiO_2_ Production

(1) Grouping and mixing ratios of specimens

One group of precursor solutions was selected. Its alkalinity was set to 6, and the available SiO_2_ solid content and water content were 0.74 g and 47.93 g, respectively. The precursor solution was prepared according to the procedure given in Section 2.2.1 and then added to the cement. The specific mixing ratio is given in Table 8. The prepared specimen is denoted as W2. W0 refers to the pure cement-based material before adding the precursor solution, and W1 refers to the cement-based material with 0.74 g of nano-SiO_2_ powder.

(2) Preparation and analysis of specimens

Fresh cement pastes of W0–W2 were put in 40 mm × 40 mm × 40 mm cubic test moulds, followed by compaction through vibration. Next, the cement pastes were cured for 15 h under standard conditions to prepare the cement paste samples. At this time, the strengths of the cement paste samples were preliminarily established, which was conducive to the preparation of samples. The hydration reaction of cement had just commenced, and the internal structure was relatively loose. This was a good time to observe the reaction products of cement hydration and nano-SiO_2_.

## 3. Experimental Results and Analysis

### 3.1. Effects of the Alkalinity of the Precursor Solution on the Compressive Strength of Cement-Based Materials

**Figure 1:**
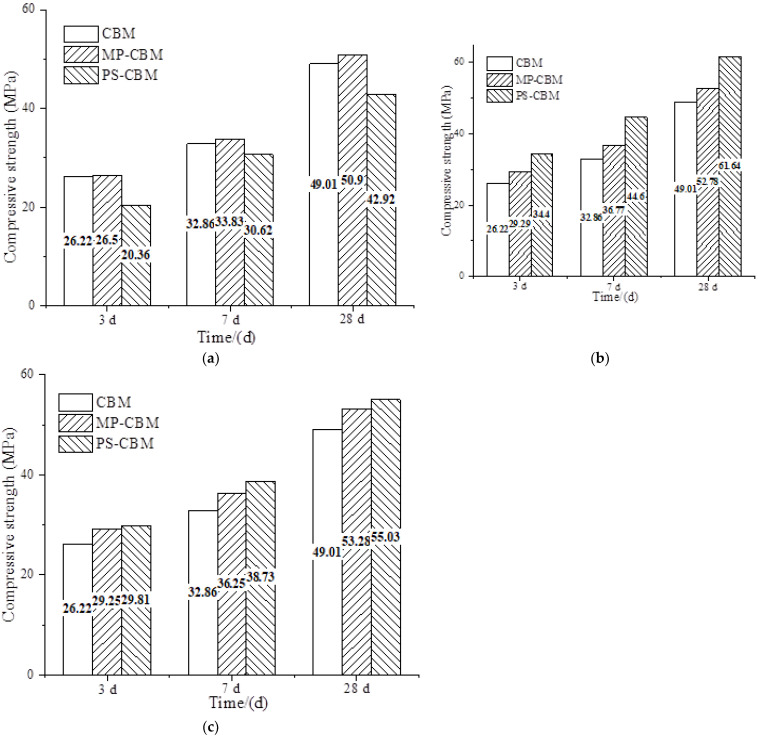
The influence of the alkalinity of the precursor solution on the compressive strength of cement-based materials. (**a**) NSPS pH = 4, (**b**) NSPS pH = 6, and (**c**) NSPS pH = 8.

The influence of the alkalinity of the precursor solution on the compressive strength of cement-based materials is shown in Figure 1, where CBM refers to pure cement-based materials, MP-CBM refers to cement-based materials with nano-SiO_2_ powder, and PS-CBM refers to cement-based materials with the precursor solution. It can be seen in Figure 1a that when the pH of the precursor solution was 4, the compressive strengths of CBM, MP-CBM, and PS-CBM increased with time. Specifically, PS-CBM had the lowest compressive strength, while MP-CBM had the highest compressive strength.

In Figure 1b, when the pH of the precursor solution was 6, the compressive strengths of CBM, MP-CBM, and PS-CBM increased with time. Specifically, the compressive strength of PS-CBM was the highest, followed by that of MP-CBM. The compressive strength of CBM was the lowest. At 3 d, 7 d, and 28 d, the compressive strengths of PS-CBM were about 17.45%, 21.30%, and 16.80% higher than those of MP-CBM, respectively. The increase was the highest after 7 d, which was about 25–36% higher than that of CBM. The compressive strengths of PS-CBM at 3 d, 7 d, and 28 d were 68.96%, 45.66%, and 43.62% higher than those when the pH of the precursor solution was 4, respectively. With the increase in age, the increasing rate of compressive strength decreased. However, the overall compressive strength of PS-CBM was higher than that of CBM and PS-CBM; the increasing rate was relatively ideal. 

It can be seen from Figure 1c that when the pH of the precursor solution was 8, the compressive strength of PS-CBM was similar to that of MP-CBM at 3 d, but it was 6.84% and 3.28% higher than that of MP-CBM at 7 d and 28 d, respectively. The compressive strengths of both MP-CBM and PS-CBM were always higher than that of CBM. The compressive strengths of PS-CBM at 3 d, 7 d, and 28 d decreased by 15.40%, 15.16%, and 12%, respectively, compared to those when the pH of the precursor solution was 6. With changes in age, the decreasing rate of compressive strength declined, and the overall decreasing rate was obvious.

### 3.2. Effects of the Available SiO_2_ Solid Content in the Precursor Solution on the Compressive Strength of Cement-Based Materials

**Figure 2:**
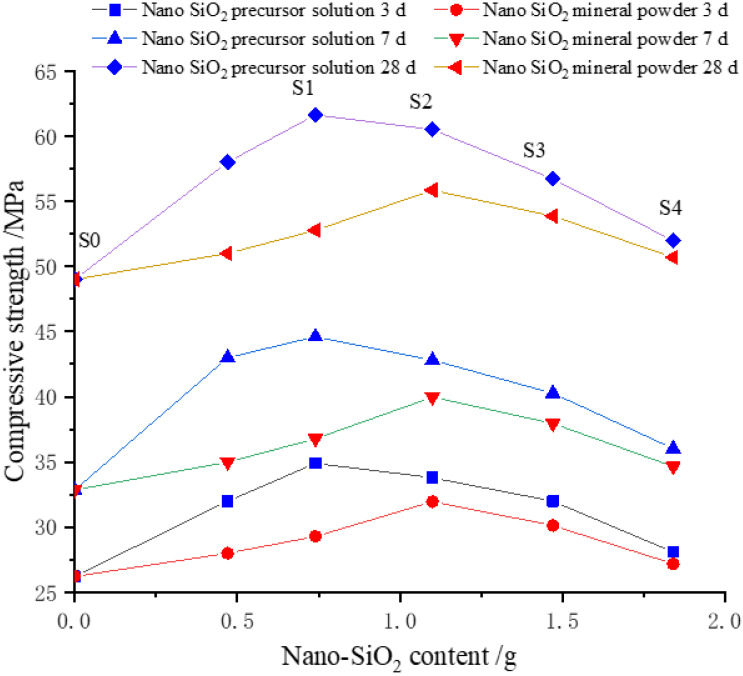
Effect of nano-SiO_2_ content on compressive strength of cement-based materials.

The effects of the available solid content of SiO_2_ in the precursor solution on the compressive strength of cement-based materials are shown in Figure 2. The blue lines, from the bottom to the top, represent the compressive strengths of PS-CBM at 3 d, 7 d, and 28 d. The red lines, from the bottom to the top, represent the compressive strengths of MP-CBM at 3 d, 7 d, and 28 d. The compressive strengths of CBM are the three values in the upper part when the available SiO_2_ solid content is 0.

It can be seen in Figure 2 that the compressive strengths of PS-CBM first increased and then decreased with increases in the available SiO_2_ solid content in the precursor solution. The compressive strengths of MP-CBM first increased and then decreased with increases in the nano-SiO_2_ powder content. However, the compressive strengths of MP-CBM were all lower than those of PS-CBM. Moreover, the compressive strengths of PS-CBM and MP-CBM were 25.78–35.73% and 7.69–11.71% higher than those of CBM, respectively.

Furthermore, the compressive strength of PS-CBM reached the maximum value earlier than that of MP-CBM. When the compressive strength of MP-CBM reached a maximum, it was lower than that of PS-CBM.

## 4. Microstructural Analysis of Cement-Based Materials after Nano-SiO_2_ Internal Production

### 4.1. Pure Cement-Based Materials

**Figure 3:**
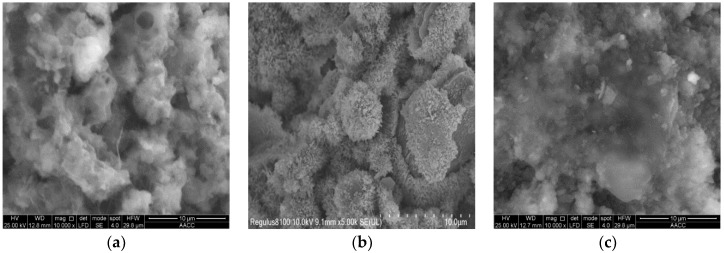
Cement-based materials of different admixtures. Reprinted from Ref. [19]. (**a**). Pure cement-based materials. (**b**). Cement-based materials with nano-SiO_2_ powder. (**c**). Cement-based materials with internal nano-SiO_2_ production.

The microstructure of pure cement-based materials after hydration for 15 h is shown in Figure 3a. In W0, there were large gaps and relatively independent pores among cement particles, an uneven cement surface, and a relaxed and porous structure. The cement particles were wrapped by layers of floccules, which covered the cement particles like layers of yarn. Moreover, many interlacing and needle-like hydration products were generated on the cement particle surface. 

### 4.2. Cement-Based Materials with Nano-SiO_2_ Powder

The microstructure of cement-based materials with nano-SiO_2_ powder after hydration for 15 h is shown in Figure 3b. There was obvious powder agglomeration among the cement particles. A layer of white hydration products—calcium silicate gel—was adhered to the agglomeration surface. The agglomerates and agglomeration layer adhered to the cement particle surface, which then filled in the gaps among cement particles. However, the agglomeration was relatively loose, and the internal structural compactness was relatively poor [25,26]. The cement particles of cement mortar with nano-SiO_2_ powder were more compact and had smaller pores than W0 [27,28].

### 4.3. Cement-Based Materials with Internal Nano-SiO_2_ Production

The microstructure of cement-based materials with internal nano-SiO_2_ production after hydration for 15 h is shown in Figure 3c. The hydration degree of cement particles was uniform. Particles were wrapped and covered by gel. The flat floccules began to wrap cement particles gradually, and particles became tightly connected. The hydration products surrounding the cement particles gradually increased, as did the thickness of the hydration layer. They filled in the spaces surrounding the cement particles and tightly connected them, increasing the compactness of the cement mortar gradually and decreasing the number of pores. In comparison to pure cement mortar and cement mortar with nano-SiO_2_ powder, cement mortar with internal nano-SiO_2_ production exhibited a more compact structure, tighter connection of the generated gel products, and smaller pores. As a result, the ability of the structural system to resist external loads was strengthened [29,30].

### 4.4. Energy Spectrum Test Results

**Figure 4:**
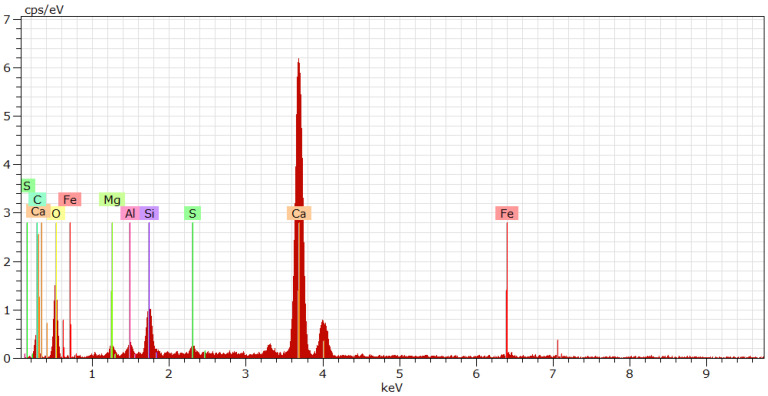
Energy spectrum near hydration product.

An energy spectrum analysis diagram of cement paste in a small area under 10,000× magnification is shown in Figure 4. The cement particles mainly contained Ca, Si, Al, and Fe, while the precursor solution mainly contained Si and O. When the cement particles just have contact with the NSPS, the surfaces of the cement particles partially dissolve, and the calcium ions in the cement particles dissolve in the water, forming a strongly alkaline solution. In an alkaline environment, not only does the active Si-O bond on the surface of cement particles break, which causes silicon to enter the solution and combine with water, but it also promotes the precipitation of H_2_SiO_3_ in the NSPS [19].

## 5. Reaction Mechanism Analysis of Cement-Based Materials with Nano-SiO_2_ Internal Production

### 5.1. Reaction Mechanism of CBM

**Figure 5:**
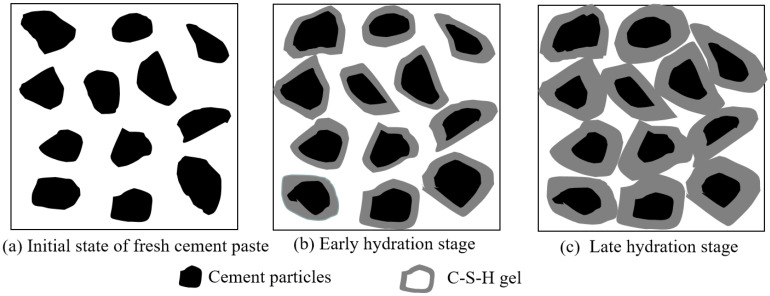
The reaction mechanism of initial state, early hydration stage and late hydration stage of CBM.

The reaction mechanism of CBM is shown in Figure 5. Cement particles that were not hydrated in the beginning remained independent. After hydration began, cement reacted with water to generate a thin layer of calcium silicate hydrate (C-S-H) gel surrounding it. As the hydration process continued, C-S-H became thicker and thicker and wrapped the cement particles tightly, which were pulled together and bonded tightly, increasing the strength of the mortar. However, there were still spaces among the particles, which influenced the strength development. 

### 5.2. Reaction Mechanism of MP-CBM

**Figure 6:**
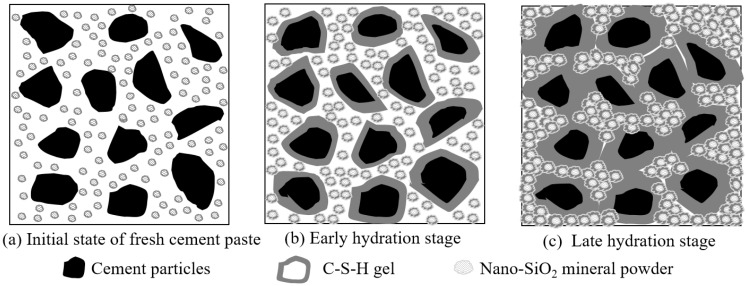
The reaction mechanism of initial state, early hydration stage and late hydration stage of MP-CBM.

The reaction mechanism of MP-CBM is shown in Figure 6. The initial state, when nano-SiO_2_ powder was added to the cement-based materials, is shown in Figure 6a. Owing to the small size, large specific surface area, and low density of nano-SiO_2_ powder particles, they agglomerated after adding them to cement mortar and stirring. The internal structure of the micro-agglomerates was as loose as flour paste, exhibiting a poor agglomeration force and cohesive force. With increases in the nano-SiO_2_ powder content, there were more floccules on the cement particle surface, and the viscosity of cement mortar increased, thus decreasing its liquidity correspondingly.

As the hydration continued, Ca(OH)_2_—which was formed from the hydration between nano-floccules and cement particles—reacted to generate C-S-H, which wrapped the floccule surfaces (Figure 6b). The addition of nano-SiO_2_ powder accelerated cement hydration. Since the generated C-S-H tightly wrapped the floccules, they were separated from external products. The nano-SiO_2_ powder in the floccules could not continue to participate in the reaction, resulting in a looser internal structure but a compact external structure of the floccules.

It can be seen in Figure 6c that, during late cement hydration, adding nano-SiO_2_ powder facilitated the large-scale generation of hydration products on cement particle surfaces. The C-S-H was thickened, and cement particles became more tightly connected. Moreover, nano-SiO_2_ powder agglomerations filled in gaps among cement particles to bind particles tighter and made them more compacted, further facilitating the development of mortar strength. However, the strength, in terms of resisting external stress, was not fully developed due to the incomplete reaction in nano-SiO_2_ powder agglomerations [31].

### 5.3. Reaction Mechanism of PS-CBM

**Figure 7:**
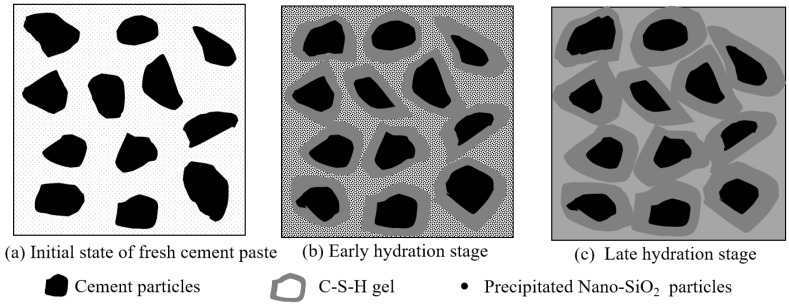
The reaction mechanism of initial state, early hydration stage and late hydration stage of PS-CBM.

The reaction mechanism of PS-CBM is shown in Figure 7. The scenario in which the precursor solution was just added to cement-based materials is shown in Figure 7a. Since cement-based materials are alkaline, nano-SiO_2_ particles in the precursor solution precipitated very quickly from the solution and dispersed uniformly in the mortar [32].

Figure 7b shows that the addition of the precursor solution accelerated cement hydration, leading to the increased generation of C-S-H. Further, cement particles became wrapped by the generated C-S-H gel. In addition, there were several active groups on uniformly dispersed nano-SiO_2_ nanoparticle surfaces in the mortar (-Si-OH), which had ultrahigh chemical activity. They underwent a pozzolanic reaction with the cement hydration product Ca(OH)_2_, generating C-S-H gel and decreasing the content of Ca(OH)_2_, which could easily corrode.

Figure 7c shows that nano-SiO_2_ particles could not only start a pozzolanic reaction but could also serve as crystal nuclei in cement mortar. The newly generated C-S-H gel bonded to the nanoparticle surfaces and changed their structural form. New networks were rebuilt based on the original network structure of hardened cement mortar [16]. These new networks filled in spaces among cement particles to increase the compactness and bonding strength among particles. Hence, the mechanical properties of cement-based materials were effectively improved.

### 5.4. Microstructural Model of PS-CBM

**Figure 8:**
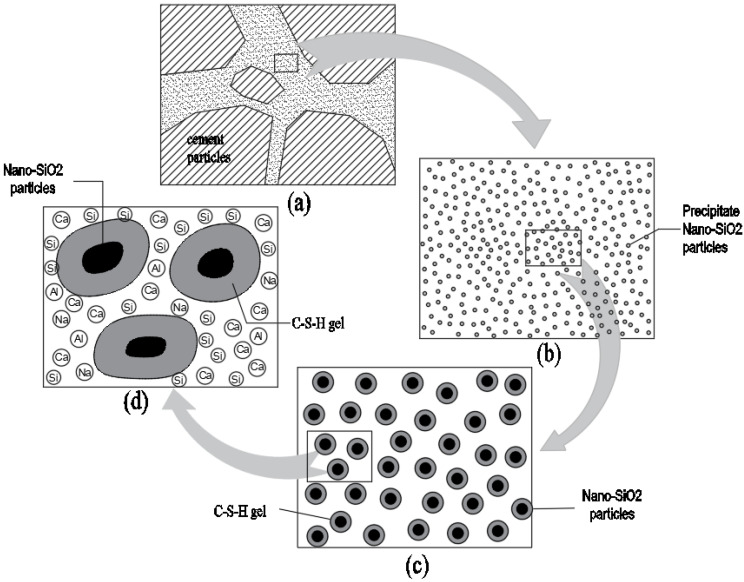
The microstructural reaction scale up model of PS-CBM.

The microstructural reaction model of PS-CBM is shown in Figure 8. The diagram of PS-CBM is shown in Figure 8a. Figure 8b is an enlarged view of Figure 8a, which mainly comprises cement particles, precipitated nano-SiO_2_ particles, and reaction products. The NSPS, prepared using the liquid-phase technique, is a slightly acid solution in which nano-SiO_2_ particles have not yet precipitated. After they were added to cement mortar, the SiO_2_ in the solution will precipitate as nanoparticles and disperse uniformly in cement mortar (Figure 8a). The precipitated nano-SiO_2_ particles underwent a pozzolanic reaction with Ca(OH)_2_ in cement-based materials to generate C-S-H gels [5]. Further, nano-SiO_2_ particles served as crystal nuclei in cement-based materials. As nodes of networks, nano-SiO_2_ particles could bond more extensively with nano-level C-S-H gels (Figure 8c). Three-dimensional network structures were formed, which changed the structural form and filled in small pores in hardened cement-based materials. As a result, the compactness of cement-based materials was improved, and their compressive strength increased.

### 5.5. Microstructural Model of Precipitated Nano-SiO_2_ Particles

**Figure 9:**
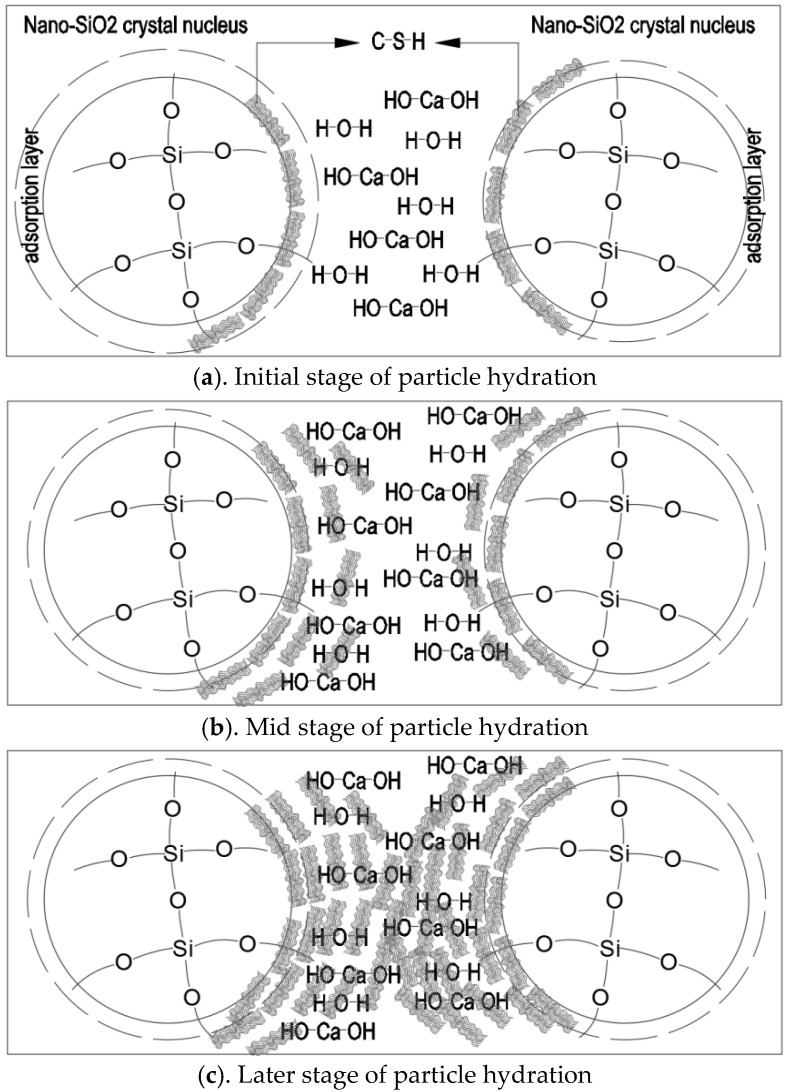
Microstructural model of precipitated nano-SiO_2_ particles.

Nano-SiO_2_ particles mainly serve as activators and reactants in cement-based materials (Figure 9). In Figure 9a, the precursor solution facilitated the hydration process of cement as an activator after it was added to cement-based materials. When the nano-SiO_2_ particles precipitated as reactants, they could react with dissolved calcium and water in cement hydration products to produce C-S-H due to their own adsorption. The produced C-S-H adhered to nano-SiO_2_ particles. Nano-SiO_2_ provided new nucleation sites for C-S-H gel as active fillers. It can be seen in Figure 9c that, as the reaction continued, more and more C-S-H gels formed around the particles. The nano-SiO_2_ particles mutually bonded to form new spatial network structures, which filled in the pores among cement particles. This decreased the number of pores, increased the cohesive strength, and improved the compactness of the base material.

## 6. Conclusions

The strengthening effects of an NSPS on cement-based materials are mainly affected by the pH value of the precursor solution and the content of nano-silica precipitated from the precursor solution. Changes in these two factors affect the mechanical strength of cement-based materials mainly as follows: (1) when the current drive solution is acidic, the compressive strength of cement-based materials after internal nano-SiO_2_ production is the largest, which is greater than that when the precursor solution is acidic or alkaline, and it is greater than the compressive strength of cement-based materials mixed with nano-SiO_2_ powder; (2) with the increase in the solid content of nano-SiO_2_ in the precursor solution, the compressive strength of the cement-based material prepared with it first reaches the highest point and then gradually decreases, but its compressive strength is always greater than that of the cement-based material mixed with nano-SiO_2_ mineral powder. The above results indicate that the precursor solution makes a great contribution to the compressive properties of cement-based materials. However, the hydration heat and hydration kinetics of the NSPS on cement-based materials also need to be further tested and simulated to study the impact of the NSPS on the entire hydration process of cement-based materials.

## Figures and Tables

**Table 1:** Chemical composition of cement.

**Ingredient**	SiO_2_	Al_2_O_3_	Fe_2_O_3_	CaO	MgO	Na_2_O	f-CaO	Loss
**Content/%**	21.85	5.62	2.99	61.55	2.64	0.44	0.92	2.53

**Table 2:** Physical performance index of Water Glass.

Main Ingredients	Modulus	Baume (°)	Moisture Content (%)	Density (g/cm^3^)	Melting Point (°C)	Boiling Point (°C)	Vapor Pressure (kPa)
Na_2_SiO_3_	3.15	38	63	2.33	1410	2355	18

**Table 3:** Chemical Composition of Water Glass.

**Chemical Element**	${\mathrm{SiO}}_{2}$	${\mathrm{Na}}_{2}O$	${\mathrm{Al}}_{2}O_{3}$	${\mathrm{Fe}}_{2}O_{3}$	CaO	$K_{2}O$	${\mathrm{TiO}}_{2}$	S
**Content (%)**	71.60	26.52	0.72	0.22	0.14	0.15	0.04	0.38

**Table 4:** Physical properties of acetic acid.

Molecular Formula	Density (kg/L)	Substance Concentration (mol/L)	Viscosity (m. Pa.s)	Specific Heat Capacity (kJ/(kg·℃))	Saturated Vapor Pressure (kPa)	Boiling Point (℃)	Melting Point (℃)
CH_3_COOH	1.05	17.14	1.22	2.08	1.52	117.9	16.6

**Table 5:** Physical Properties of Nano-SiO_2_ Powder.

Surface	Whiteness	Average Grain Diameter (nm)	Specific Area (m^2^/g)	Density (g/cm^3^)	Loss on Drying (%)	Melting Point (℃)	Element Content (%)
White powder	94.7	≤20	600	2.6	5.1	1610	SiO_2_ ≥ 99.9

**Table 6:** The ratio of precursor solution with different alkalinity levels.

Number	Nano-SiO_2_ Precursor Solution				Cement/g	Water/g	Sand/g
	Acetic Acid/g	Sodium silicate/g	pH	SiO_2_ Solid Content/g			
J0	/	/	/	0	450	225	1350
J1	40	1.70	4	0.11	450	184.12	1350
J2	40	11.11	6	0.74	450	177.07	1350
J3	40	12.33	8	0.82	450	176.15	1350

**Table 7:** Cement-based material ratio of precursor solution with different nano-SiO_2_ content.

Number	SiO_2_ Solid Content/g	pH	Water/g	Cement/g	Sand/g
S0	/	/	225	450	1350
S1	0.74	6	177.07	450	1350
S2	1.10	6	153.10	450	1350
S3	1.47	6	129.14	450	1350
S4	1.84	6	105.17	450	1350

**Table 8:** Specific mixing ratio.

Number	SiO_2_ Solid Content/g	pH	Nano-SiO_2_ Powder/g	Water/g	Cement/g
W0	/	/	/	225	450
W1	/	/	0.74	225	450
W2	0.74	6	/	177.07	450

## Data Availability

Not applicable.
